# Supporting wellness after cancer treatment for women from Chinese, Vietnamese, and Arabic backgrounds: a qualitative study of healthcare provider views

**DOI:** 10.1007/s00520-025-09417-6

**Published:** 2025-04-17

**Authors:** Suzanne Grant, Tinashe Dune, Elisabeth Elder, Jolyn Hersch, Cannas Kwok, Judith Lacey, Eric Yeung, Kylie Barr, Nema Hayba, Ash Malalasekera, Joel Rhee, Carolyn Ee

**Affiliations:** 1 NICM Health Research Institute, Western Sydney University, Locked Bag 1797, Penrith South, DC NSW 2751 Australia; 2 Translational Health and Research Institute, Western Sydney University, Locked Bag 1797, Penrith South, DC NSW 2751 Australia; 3 Western Sydney University, Locked Bag 1797, Penrith South, DC NSW 2751 Australia; 4 Westmead Breast Cancer Institute, Westmead Hospital, 166 - 174 Darcy Road, Westmead, NSW 2145 Australia; 5 Westmead Clinical School, University of Sydney, Cnr Hawkesbury Road and Darcy Rd, Westmead, NSW 2145 Australia; 6 Sydney School of Public Health, Faculty of Medicine and Health, Sydney Health Literacy Lab, the University of Sydney, Sydney, NSW 2006 Australia; 7 School of Nursing, Paramedicine and Healthcare Sciences, Faculty of Science and Health, Charles Sturt University, Panorama Avenue, Bathurst, NSW 2795 Australia; 8 Department of Supportive Care and Integrative Oncology, Chris O’Brien Lifehouse, Camperdown, NSW Australia; 9 Clinical School of Medicine, The University of Sydney, Sydney, NSW 2006 Australia; 10 Canrevive, 4/741 George St, Haymarket, NSW 2000 Australia; 11 Bankstown Cancer Centre, South Western Sydney Local Health District, Sydney, NSW Australia; 12 School of Clinical Medicine, Discipline of General Practice, Faculty of Medicine and Health, UNSW Sydney, Sydney, NSW 2052 Australia

**Keywords:** Ethnic minorities, CALD, Survivorship, Cancer, Language

## Abstract

**Introduction:**

Over one million Australians live with a cancer diagnosis, with nearly a quarter speaking a language other than English. Cancer survivors from culturally and linguistically diverse (CALD) backgrounds often face significant unmet needs during survivorship, including navigating the healthcare system and accessing culturally appropriate support. For example, Chinese- and Vietnamese-speaking survivors report physical and psychosocial impacts, compounded by limited availability and access to tailored information on symptom management and recurrence prevention.

This study aimed to explore healthcare providers’ perspectives on designing supportive care programs for women cancer survivors from Vietnamese, Arabic, and Chinese-speaking backgrounds, focusing on culturally appropriate content, delivery formats, and barriers and facilitators to engagement.

**Method:**

Thirteen healthcare providers experienced in cancer survivorship and supportive care for CALD women participated in semi-structured interviews. Participants were recruited from healthcare settings in Western Sydney, a culturally diverse region, using purposive sampling to ensure diverse professional perspectives. Interviews were guided by an advisory committee, audio-recorded, transcribed verbatim, and analyzed thematically using NVivo.

**Results:**

Three key areas were identified: program content, delivery preferences, and barriers/enablers. A holistic approach addressing physical, emotional, and social dimensions was recommended, incorporating culturally tailored guidance on diet, exercise, and mindfulness. Non-hospital, community-based settings were favored for accessibility and comfort, with a stepped-care model offering varying levels of support based on individual needs.

Challenges included language barriers, privacy concerns, and logistical issues, while facilitators encompassed culturally sensitive outreach, community partnerships, and bilingual facilitators. Participants emphasized the importance of low-cost programs with flexible delivery formats.

**Conclusions:**

This study provides valuable insights from healthcare providers on the design of culturally and linguistically tailored supportive care programs for women cancer survivors from Vietnamese-, Arabic-, and Chinese-speaking backgrounds. Providers emphasized the importance of a holistic approach addressing physical, emotional, and social needs, with delivery in accessible, community-based settings. Key recommendations included culturally sensitive outreach, bilingual facilitators, and flexible, low-cost program options to overcome barriers such as language, privacy concerns, and logistical challenges. These programs have the potential to advance health equity by improving survivorship experiences and outcomes for culturally diverse women.

**Supplementary Information:**

The online version contains supplementary material available at 10.1007/s00520-025-09417-6.

## Introduction

Over 1 million Australians live with a cancer diagnosis [1], and almost a quarter of these cancer survivors speak a language other than English [2]. People from culturally and linguistically diverse (CALD) backgrounds have greater unmet cancer supportive care needs in survivorship compared to Anglo-Australian individuals, and report difficulty navigating the healthcare system [3, 4]. For example, individuals from Chinese- and Vietnamese-speaking backgrounds report the experience of having cancer to be distressing and isolating [5], with substantial physical and psychosocial effects persisting years into survivorship [6].

Supportive care in cancer refers to the prevention and management of adverse effects of cancer and its treatment, and includes management of physical and psychological symptoms and side effects across the continuum of the cancer journey [7]. The benefits of physical activity and a healthy diet after cancer are well established [8] and interventions such as mindfulness and yoga improve distress, anxiety and depression [9]. Despite the greater unmet needs of cancer survivors from CALD populations, the supportive care information and guidelines in Australia are primarily aimed at individuals from English-speaking backgrounds, overlooking the specific needs of culturally and linguistically diverse groups [10]. As a result, individuals from CALD populations in Australia have expressed substantial unmet needs and barriers to supportive care such as difficulty finding information on how to self-manage ongoing symptom burden from cancer treatment and reduce risk of recurrence [11, 12]. The stigma and shame of a cancer diagnosis are additional barriers to accessing adequate supportive care [13]. These disparities are particularly marked in individuals who identify as women. For example, cultural norms may impact on the acceptability of women from some CALD backgrounds engaging in physical activity [14].

In Australia, some of the most common languages spoken by migrants are Mandarin (2.7%), Arabic (1.4%), Cantonese (1.2%) and Vietnamese (1.3%) [2]. In some areas, such as Western Sydney, these groups make up a large proportion of the population. There is a significant lack of inclusion of these language groups in current cancer survivorship initiatives, and women from these CALD backgrounds have expressed substantial unmet supportive care needs [14, 15]. Yet, there is a marked paucity of research on supportive care for CALD women with cancer in Australia, and very few, if any, programs exist that have been co-designed with consumers to ensure the delivery of culturally appropriate supportive care.

In Australia, the limited supportive care in cancer services available are delivered in a range of settings including public and privately funded hospitals, general practices/primary care settings, and community health settings. Most interventions—including complementary therapies and select allied health services—incur out-of-pocket costs for patients. However, a significant gap remains in services tailored to women from Arabic-, Chinese-, and Vietnamese-speaking backgrounds. Existing programs predominantly cater to English-speaking populations, leaving culturally and linguistically diverse (CALD) groups underserved. This study addresses this gap by examining healthcare providers’ perspectives on designing culturally appropriate supportive care programs for these communities.

This paper focuses on healthcare providers’ perspectives because their insights into systemic barriers and practical implementation assist in designing feasible supportive care programs. Providers bridge the gap between patient needs and service delivery realities hence their input is necessary when developing a program of this nature. Consumer perspectives (women with cancer from CALD backgrounds) were also collected and these will be reported in a separate publication. Our research question was:

How do healthcare providers perceive the key components, delivery models, and implementation challenges of culturally tailored supportive care programs for these populations, with particular attention to stepped care approaches?

## Methods

### Study design

This qualitative study used in-depth semi-structured interviews to inform the design of a supportive care program for Australian women from Chinese-, Vietnamese-, and Arabic-speaking backgrounds. Input on study design was sought from a study advisory committee consisting of consumers with lived experience from Chinese-, Vietnamese- and Arabic-speaking backgrounds and key stakeholders. Ethical approval was granted from the Western Sydney University Human Research Ethics Committee (HREC) (H14752) in February 2022. This study is reported in accordance with the Consolidated Criteria for Reporting Qualitative Studies (COREQ) [16].

### Participants

Participants were healthcare providers with experience in cancer survivorship and supportive care for women from Arabic-, Chinese-, and Vietnamese-speaking backgrounds. They included providers based in community settings, cancer centres, and primary care. Eligibility criteria required participants to have specific qualifications or experience in supportive care, with at least one of the target populations. We used community organisations, social media, and word of mouth to recruit. Purposive sampling aimed for diversity across healthcare disciplines.

### Recruitment and sampling

Data collection and data analysis occurred concurrently to determine the point at which data was considered adequate in terms of richness and complexity in order to answer our research questions [17]. This process guided the determination of our final sample size. A study advisory committee recommended key stakeholder organisations and healthcare providers within the Western Sydney area as recruitment starting points. Stakeholder organisations and HCPs were emailed the Participant Information Sheet with an embedded REDCap weblink, and invited to register their interest in the study. A member of the study team contacted the participant by phone or email to answer any questions and arrange an appointment time for a focus group/interview. Written consent was obtained electronically using REDCap. Consent was also verbally confirmed prior to commencing the recording of focus groups/interviews.

### Data collection

Semi-structured interviews were conducted by researchers with experience in qualitative methods (SG, NH). All interviews were conducted in English. An interview guide was developed by research team and the advisory committee. The interview guide explored the experience of HCPs working with people with cancer from Vietnamese-, Arabic-, and Chinese- speaking backgrounds, and preferences for a culturally sensitive supportive care program (see Supplemental File 1).

The study adopted an exploratory approach to investigate healthcare providers’ perspectives on supportive care program design, with particular interest in the stepped care model. While a preliminary 8-week program framework was visually introduced during interviews, this occurred only after initial open-ended discussions to capture novel insights. The proposed program was presented as a flexible, loosely constructed concept specifically to stimulate discussion rather than limit responses. The preliminary 8-week program was developed by the research team based on literature, and meetings with the advisory committee (see Supplemental File 2). Participants were advised that the research team intended to pilot a program following this co-design phase. Participant views and experiences were elicited to determine potential cultural sensitivities that may influence the intervention delivery content and format as well as potential barriers and facilitators to engaging in a supportive care program. Interviewers were two experienced researchers, both held a PhD, had formal training in qualitative research methods and were female. Two study participants were known to the researcher (SG) through previous engagement in supportive care in cancer activities. One of the interviewers (NH) was from an Arabic-speaking background, and had no prior relationship with any of the study participants.

Interviews lasted for between 30 and 60 min, were conducted in English and were digitally recorded (audio and video) using *Zoom* and transcribed verbatim using Trint [18]. One interview was conducted by telephone and audio recorded only. Transcripts were checked against the original recording, corrected and deidentified. Transcripts were not returned to study participants for correction. Demographic data was stored in *REDCap*, and deidentified transcripts were stored on the Western Sydney University secure server. To compensate participants for their time, we offered gift vouchers to the value of $100 AUD to HCPs who provided data in this study. Participants could choose to decline the voucher.

### Data coding and analysis

Transcripts were analysed thematically [19] by SG, CE, and an independent research assistant (KB) using *NVivo* [20]. Throughout this process, SG and KB met regularly to discuss and iteratively refine the analysis. The qualitative research methodology used in this study is thematic analysis, as outlined by Braun and Clarke [19]. This approach was chosen for its flexibility in identifying patterns and themes within the data, allowing for both deductive and inductive analysis. Themes were preselected based on the specifics of the research question (deductive), and sub-themes generated from the data through open coding (inductive).

Transcripts were read once, and audio recordings reviewed to reach immersion. Reflexive notes were written on hard copies of the transcripts to record key ideas arising from responses. A deductive thematic map was sketched, derived from the main areas of the interview guide designed to answer the research question: program content, program delivery, and barriers and facilitators. This was expanded/supplemented with sub themes that we identified by inductive analysis from the data set. A linear version of the map was then prepared as an initial code list with descriptions (i.e., operational definitions) for entry and application in NVivo qualitative research software. This process also involved further refinements to the coding structure where codes were collapsed into themes (and related sub-themes) aligned with the interview guide.

Transcripts were coded electronically in NVivo wherein codes were applied to dialogue based on a natural fit with the operational definition. The last step involved tabulating a selection of exemplar quotes in Microsoft Word alongside operational definitions to illustrate each theme and subtheme.

## Results

### Participant characteristics

Of the 18 HCPs invited to participate, 13 took part in the study (12 interviews total, including one joint interview with two HCPs). Three declined due to scheduling conflicts and two did not respond. Participants included six hospital-based and seven community-based practitioners from diverse disciplines: cancer nursing (*n* = 2), physiotherapy (*n* = 2), medical professionals (*n* = 5: medical oncology, radiation oncology, and general practice), and allied health/complementary therapists (*n* = 4: counsellors, social workers). All worked in cancer care services within Western Sydney, Australia—a culturally diverse region (see Table 1).

**Table 1:** Demographic characteristics of health care professionals

Characteristic	*N* = 13
Female/Male	12/1
Occupation:	
Oncology nurses	2
Physiotherapists	2
Oncologists	2
GP	1
Allied Health + Other	6
Ethnicity*	
Australian	7
Chinese	2
Arabic	2
Vietnamese	1
Eastern European	1

*Ethnicity defined as self-identified cultural background

### Themes

**Fig. 1:**
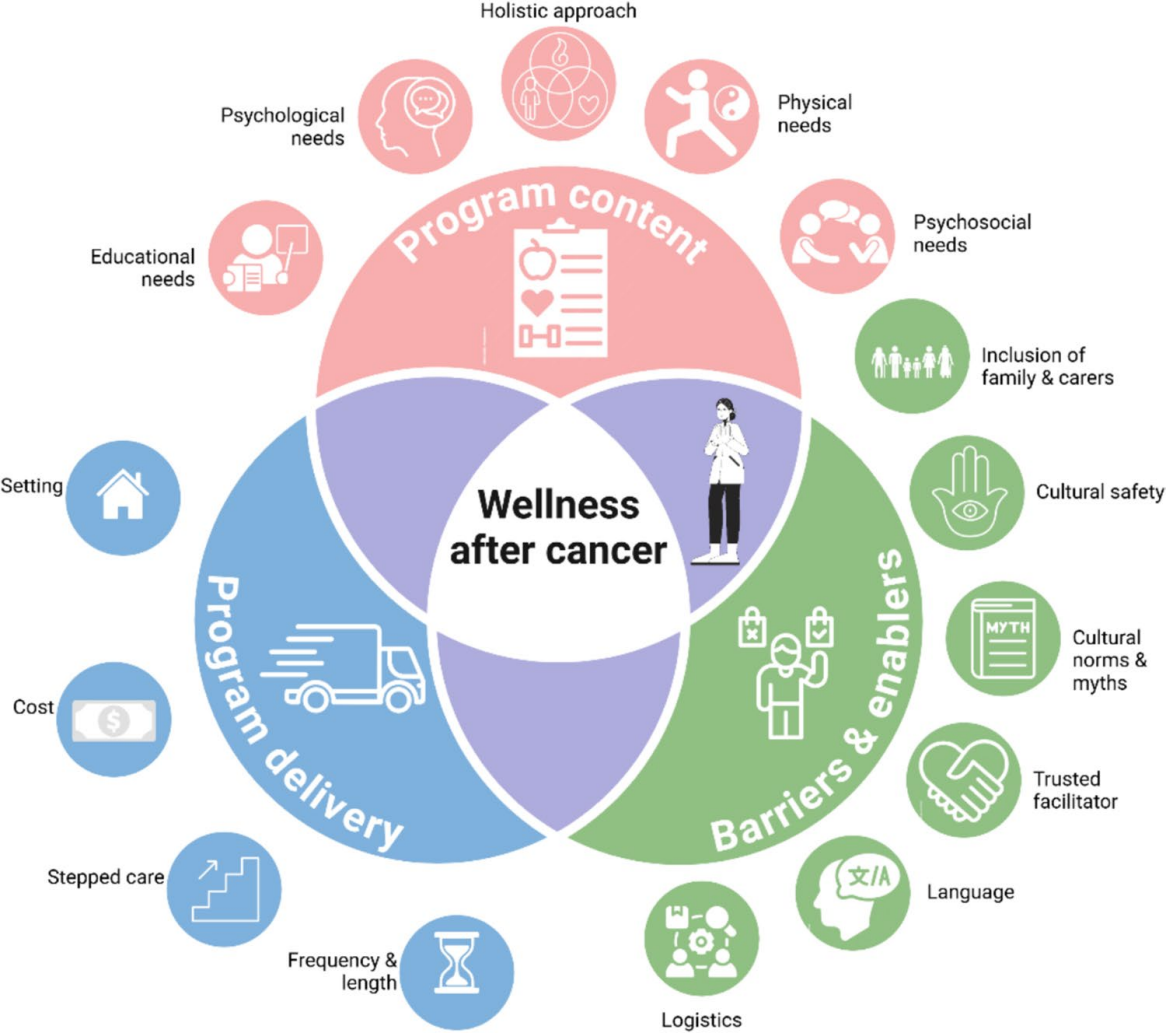
Healthcare providers themes and subthemes

Healthcare providers provided detailed insights into preferences, facilitators, and barriers concerning the design of supportive care programs for women with cancer from Arabic-, Chinese-, and Vietnamese speaking backgrounds. Three primary themes provide the framework for organizing the findings: program content, program delivery, and barriers and facilitators. Figure 1 provides a summary of themes and sub-themes.

### Program content

Participants provided their perspectives on recommended content of the program, within subthemes of *Holistic Approach; The Body – Physical Needs; The Mind – Psychological Needs; The Spirit – Psychosocial Needs;* and *The Intellect – Educational Needs.*

### Holistic approach

Many healthcare providers spoke about the need to take a holistic approach, integrating physical, emotional, and social dimensions of health to support individuals in managing the impacts of cancer and its treatment, aiming to improve the quality of life for patients and their families. There was strong support for programs that covered diet, exercise and risk factors but also:

…other sort of wellness things like mindfulness, stress… and other things like Centrelink^1^ [welfare system] and financial support, things like access to superannuation…sexual health… [HCP6]

A program that transitions them back to life after cancer, combining education about expected health changes, healthy living tips, and practical activities like yoga or dancing would be ideal, especially if conducted in their native language [HCP11]

Some healthcare providers also thought that the program content would be best driven by the participants:

So I guess I think that really needs to be sort of designed by the women themselves or input at least with other professionals in saying what would be important to cover for a program like that [HCP4]

### The Body – Physical Needs

HCPs recommended meeting physical health needs of program participants by incorporating elements such as diet advice, exercise, self-massage, and health advice about managing physical issues such as lymphoedema, weight gain, pain management and fertility after cancer. These sessions should be presented by a bilingual healthcare professional. Gentle forms of physical activity that also focussed on strength and flexibility as well as providing some aerobic activity were suggested, such as yoga, dance, or *qi gong.*

But because of the limited movements that a lot of them are having, I think something that includes increase in flexibility is a very good form of exercise. So yoga, I would highly recommend [HCP7]

Addressing cultural preferences in dietary advice and catering was considered crucial to providing culturally sensitive supportive care. Some HCPs advised that programs within these CALD communities often included food as a central element, highlighting its importance in care provision.

I think ... a culturally appropriate dietitian, you know, I talk to the [CALD] women about high protein, high calorie foods and then 'do you like cheese and ice cream and dairy?'. And they’ll say 'no I don’t eat any dairy'. So [I] try to educate them about the types of products that have a lot of calcium. And even different foods because there’s no point in telling them to eat bread and cheese if they’ve never eaten them in their lives [HCP 11]

### The Mind – Psychological Needs

Healthcare providers (HCPs) emphasized the need for psychological support to address significant mental health concerns following cancer treatment, such as anxiety, depression, fear of recurrence, and issues related to body image and physical changes. Culturally sensitive psychological support should also consider the effects of shame and stigma, fears surrounding a cancer diagnosis, and concerns about potential genetic implications for their children. For some, beliefs associating cancer with karma also need to be addressed, as they may influence the individual's experience and perception of the illness,

Yeah it’s the worry that’s why and, you know, from the group, the people have to learn about some myths and beliefs about a cancer. About some true and some not true. Keep the people feeling safe and relaxed and that their life is ok now. And not think about some myths and beliefs that aren’t true [HCP 3]

In the health system, in the cancer treatment that I know of, they don’t take care of the mind very much. They only take care of the body. Whereas in my culture, mind and body are completely connected. They cannot be divided. We even say that in my [CALD] tradition that if your mind is overwhelmed, it will crush your body [HCP12]

### The Spirit – Psychosocial needs

The opportunity to meet in a group to share and connect was seen as powerful, but it was important that these group events created a sense of trust and safety. Connecting over food and creating opportunities for fun activities such as going on a picnic, were seen as culturally appropriate ways to cultivate a beneficial group environment. The benefits of meeting these psychosocial needs were seen as significant.

The groups for the people who survive with cancer…they feel more comfortable, more happy, and they feel less alone...the women found they are in the same situation [HCP3].

### The Intellect – Educational Needs

HCPs recommended that women be provided with education about the development and maintenance of a healthy lifestyle for wellbeing post cancer, coping strategies, how to navigate the healthcare system and how to access appropriate services. Education on these topics would build self-efficacy, health literacy and empowerment.

Otherwise patients just feel, I'm very busy. I have to go home. I have to look after my husband. I have to look after my kids. They are my first priority, not myself. I'm the second. And so in that way, we have to teach patients their health actually is number one, only when they are well they can look after others better in a better way [HCP2]

There was caution expressed about not overwhelming women with information, recognising the impact of the trauma of the cancer on the person with participants recommending keeping initiatives experiential with social connection and empowerment as a focus prior to diving into education too early.

And the complaint was that my brain is already too full. I just can't take in any more information. They don't want any more educational programs because they're already overwhelmed. And overwhelm is a symptom of trauma. I know education is important, but the key is to get them into a receptive state. And that requires working with the nervous system… into the social engagement …before they're even receptive to educational programs. And I think the mistake that we have made in general is funding educational programs without first addressing the trauma...educational programs need to come after. You know, they're in a comfortable, relaxed, feeling safe [HCP7]

### Program delivery

Participants described ideal program delivery within the subthemes of *Setting; Stepped care; Frequency, Length and timing* of sessions; and *Cost.*

### Setting

The importance of non-hospital settings was emphasised, as these create a more relaxed atmosphere, thought to be crucial for emotional comfort and engagement. It was generally agreed that CALD individuals with cancer did not want to return to tertiary hospital facilities where they had been treated for their cancer.

If we had programs offsite at health care facilities, you probably get a lot more people engaged. A lot of people don't want to come back to where they got bad news with a cancer diagnosis, where they had treatment and bad memories [HCP FG1]

Participants had mixed views about using an online platform for delivery. Some HCPs thought this was a good option, while others were concerned about those who are not technologically literate or do not have access to the necessary devices.

A lot of the women weren't very technical... don't even use email [ID7]

So they only had their phone, they didn't have a laptop and often times don't even use email. So I think if you went online, you would get those which were confident with online modalities, but you would lose a lot of the disadvantaged women who either can't speak English, can't read English, don't know how to operate a computer, a little bit older, and don't know how to troubleshoot when the sound is not working, you know, something like that...it depends which group you’re going for [HCP7]

Others felt the COVID- 19 pandemic had pushed everyone “online,” and that “we're all pretty tech savvy when it comes to getting online and shopping online. So offer an online program if you can't make face to face or stream it live on the day or something” [HCP FG1].

A hybrid approach of both face-to-face and group delivery was also suggested.

For some patients, remote sessions might be necessary, while others benefit more from face-to-face interaction where they can receive support from peers in a communal setting [HCP13]

### Cost

Most HCPs suggested that the program ought to be provided to consumers at no cost or marginal cost supported by government or other grants and funding, but that adequate, continuous and secure funding and support was essential to consider for sustainability of supportive care programs.

they'd pay a small amount of money. Or even if you had a sliding scale. You know, 5 to $10, ten maximum [HCP7]

Equity in access, I guess, is the other thing…Cost of the program might be a problem [HCP13]

### Stepped care

Healthcare providers were shown a visual representation of a program incorporating a stepped care model. In this model, the intensity of interventions is adjusted to meet each patient’s specific needs. Patients start with less intensive interventions, while more intensive options are available for those with higher or unmet needs. HCPs were unanimous in their support for this model. Although some HCPs pointed out that any screening at the initial program session may be challenging, as there would not be initial trust or rapport between the program participant and the person screening.

### Frequency, length, and timing of sessions

Program sessions of two hours each were seen as ideal, running for at least six weeks duration. Other considerations were the time of day and staying within the school term. While offering a program within working hours may not allow employed individuals to attend, after hours engagement was also thought to be low. Offering a range of options was suggested, to improve access and flexibility.

So I found anything over 2 hours is too long. So I think that 2 hours is the golden timeframe to run these programs, but then depends on the patients and if they're working [HCP FG1]

*Barriers and enablers*

Participants spoke of potential barriers and enablers of delivering the program.

### Cultural safety

Culturally safe and sensitive care was a consideration throughout by all participants. Creating a safe, supportive and inclusive environment was crucial for engaging CALD women in supportive care programs.

I would do it in the community, in the community center, where they feel safe, where they feel that they won't be discriminated in any way and being delivered by people who are empathetic and who really understand the nuances of the cultural things of the group... I will not do it in the hospital environment [HCP12]

Participants felt that the needs of culturally diverse communities were generally not being catered to with existing services. A few HCPs called for the establishment of culturally specific clinics, where CALD women could receive tailored services under one roof, including access to interpreters.

And then wellness centers that can cater to the cultural needs of our community. So again, I don't have a good sense of, you know, which patients from the different cultural backgrounds that I see are accessing the services at wellness centres. And I'm going to generalize here, but I suspect it's going to be tend to be more of that sort of Anglo-Saxon background patients that are going [HCP11]

The Vietnamese community... not enough support, [not] enough resources [HCP3]

A culturally specific survivorship clinic where they're seen by all the right people, in one clinic with good interpreters would significantly improve care [HCP10]

The need for gender-specific care was emphasized for women from certain cultural backgrounds, such as those belonging to Muslim communities, where there is a preference for gender segregation in care settings.

For women who've had treatment in hospital... especially when you're talking about certain cultures there needs to be a separation of men and women. So if you had men and women mixed in together that could be a barrier for a lot of the women from certain cultures. Like the Muslim background of cultures [HCP7​]

The importance of adapting supportive care practices to align with the cultural and religious beliefs of participants is emphasized. One participant detailed how they introduced *qi gong* to their Arabic-speaking group by framing it within their cultural and religious context, thus making it more acceptable and aligning with their privacy and cultural values​​.

Education and training in cultural competence for healthcare providers were seen to be essential components of culturally safe care.

It's imperative that our team understands the cultural dimensions of health, which is why we have ongoing training in cultural competence. This helps in building trust and better communication with our patients [HCP5]

### Cultural norms and myths

Cultural myths and fears about cancer diagnosis were seen to be potential barriers to engagement. HCPs were aware of cultural or traditional beliefs that cancer is contagious; secrecy and desperate need for privacy about one’s diagnosis and treatment.

Women who have cancer, keep it quiet, they are worried that their daughter won’t get married, people will think that the daughter has inherited the cancer from them – so they keep it in private” [HCP4]

HCPs reflected that privacy concerns meant avoiding further engagement with cancer-branded services, with a preference not to use ‘cancer’ in the name of a program.

Vietnamese and Chinese are quite similar... they sometimes withdraw. They don't want to participate in any program at all. They keep it to themselves [HCP2]

Reluctance to engage in additional services was partly attributed to fears that increased attendance might suggest their condition remains unresolved, thereby exacerbating concerns about their health status. Participants also expressed the apprehension of people from certain CALD backgrounds have around others within their communities might learn about their diagnosis.

Healthcare providers observed a cultural inclination to underreport symptoms or concerns, which subsequently limited engagement in health-related activities and services:

I actually think that they tend to - people from that [Chinese] community tend to minimize symptoms more because I think there's a great fear that you're going to reduce quality during treatment, you're going to reduce their dose or you're going to stop the treatment [HCP11]

Limited time is perceived as a significant obstacle, preventing individuals from participating in supportive services, particularly when compounded by a lack of familiarity with these services due to cultural background.

Barrier would be time. They probably they feel that, that they don't have time yet to do these kind of things. And so the real time and also probably lack of understanding of what we can provide to them. Because for a lot of cultural backgrounds we normally don't have these kind of services in our own country at all [HCP2]

### Skilled and trusted facilitator

For effective program facilitation, trust and cultural alignment between facilitators and participants were identified as crucial elements. Most HCPs stated that program facilitators who share similar cultural backgrounds and language with participants may significantly enhance comfort and engagement, creating a more supportive environment that fosters open communication.

One participant emphasized the importance of trust-building measures, stating:

I guess you need to gain their trust in a way don't you. I mean, I guess by telling them the things that empower them, that this is confidential, you're in a safe environment. We're here to help. We're here to assist you. Now can you tell us, those things that perhaps allow them to feel comfortable to divulge information to you or get to the real crux of whatever this issue is that they have. I guess that's important. So I guess just building a rapport, making them feel comfortable. I mean, I guess really delving down into their own culture and what is acceptable to them [HCP13]

Similarly, healthcare providers noted that facilitators with cultural and linguistic commonalities would be beneficial:

If you could have a program that was led by someone from the same background and culture and the same language group I think that would probably be a really big bonus [HCP11]

This underscores the need for facilitators who reflect participants’ backgrounds, enhancing relatability and breaking down barriers to engagement.

### The role of cultural competence

A lack of cultural competence was recognized as a potential barrier, with providers highlighting the need for facilitators to be both trauma-informed and sensitive to cultural nuances. As one provider noted,

A lack of cultural competence can severely impact the effectiveness of the program. Healthcare providers need to be trauma-informed and sensitive to the cultural backgrounds of the patients [HCP7].

An ideal facilitator, therefore, would not only have a medical, nursing, or other health background with experience in oncology but would also be able to engage with participants in a way that respects and incorporates their cultural beliefs and practices. Attention to group composition and dynamics was also recommended to ensure participants felt comfortable and could relate to one another, avoiding disengagement due to interpersonal or cultural conflicts. A culturally attuned approach, and sensitivity was seen as important in building meaningful connections in a supportive care programs.

### Language

Being unable to converse fluently in English was clearly identified as a barrier to engagement in programs conducted in English.

...we can do it by Vietnamese…they want to talk, they want to share, because they understand the culture - the Vietnamese culture [HCP1]

..language first, first and foremost comes to mind. Language barriers. So having bilingual leaders or facilitators is very important [HCP7]

There was a reluctance to use interpreters for assistance for fear of loss of privacy about health in smaller close-knit communities. Even when interpreters are used, medical details could be misinterpreted or not translated accurately. Communication difficulties also stemmed from cultural tendencies to minimise symptoms.

And sometimes I think even with an interpreter, it can be hard to explain sort of symptoms or the reasons behind them and how to manage them and what to expect. Even with an interpreter on board [HCP11]

### Inclusion of families and carers

The inclusion of family and carers in supportive care programs was emphasised due to the high importance placed in non-Western cultures on the family unit as opposed to the individual.

Yeah. I think that probably family is the big thing with a lot of those cultures and potentially it's how we draw in some of the family to help support the women, in particular to take on lifestyle changes. I think that in some of those cultures the family's not as present in the consultations, and so it's often something we overtly have to invite them in and say, Hey, who have you got in your family that you want to bring along to this? Because it will help us making those discussions and those choices [HCP4]

“Care for carers” was viewed as important in order to support people who are assisting their family member to recover from cancer. Families and carers were also often perceived as essential to assist with language difficulties.

So I think it's important to involve the carers as well and I think they're a bit forgotten about at times. They just do all the hard work and all that stress, worrying about appointments and getting people in treatment and so forth. But they've been neglected when it comes to our services [HCP5]

### Raising awareness about the program

A lack of awareness about available programs emerged as a significant barrier to participation. Participants noted that people often remain unaware of supportive services unless they actively seek out information. As one healthcare professional stated,

I think sometimes that too, that people don't know what's out there until they actually start asking or research for themselves [HCP13]

This highlights the need for proactive efforts in promoting programs to ensure they reach those who could benefit most.

It was recommended that program advertising be culturally relevant, sensitive, and specifically targeted. Engaging with key figures within CALD communities, including respected opinion leaders, as well as spiritual and religious leaders (such as Muftis and Priests), was emphasized as essential. Utilizing faith-based institutions (temples, mosques, churches), community newspapers, in-language radio stations, social media groups, and dedicated websites were all suggested as effective methods for raising program awareness. Additionally, oncology teams and primary care networks were seen as valuable referral sources to help inform patients about available supportive care options.

I think they should get it from their oncologists. Just before the oncology treatment finishes or start talking in midway. You know, when you finish the treatment, this is something that you can do to take care of yourself, I think that should be drummed in right through until they finish and then, boom, there it is [HCP12]

Another participant highlighted the value of involving community leaders, stating,

The programs across the district, if you can engage with community leaders or elders, it has big advantages. They have more sway in the communities than what we do as health professionals [HCP FG1].

Engaging these trusted figures can increase program credibility and encourage participation from within the community.

### Logistics

Logistical challenges such as transportation and scheduling conflicts were mentioned. Poor physical health (including pain, fatigue, and other symptoms and side effects) post treatment was mentioned as inhibiting attendance and engagement in programs.

Elderly patients might have mobility issues, making it hard to participate without adequate transportation options [HCP FG1]

I mean, when you break it right down, do these women drive, how do they get to these facilities? Transport can be a problem [HCP13]

Features about the venue and its location that participants felt may not be convenient or attractive to consumers included: limited car parking spaces; cost of parking; lack of public transport services; large walking distance from parking and public transport to facilities; and not wanting to return to a medical environment.

### Financial barriers and sustainability of programs

Financial barriers were a concern, especially for programs not fully funded or requiring participants to bear some cost. This issue is exacerbated for individuals from lower socioeconomic backgrounds.

Most of them tend to be disadvantaged and it would need to be a funded project [HCP7]

HCPs spoke of programs that were popular but did not continue due a lack of ongoing funds. Therapeutic bonds and relationships formed among consumers within group programs are then severed when funding runs out and groups are disbanded.

But after that she was really sorry because after that the funding was finished. That's why we can't continue and the women cried and they say, oh yeah and we really, really wanted to, you know, to get funding to support because I always try to help feel them more comfortable and give them all information about health and wellbeing for them [HCP3]

## Discussion

This study explored HCPs perspectives on preferences for supportive care program design for women with cancer from Chinese, Vietnamese, and Arabic speaking backgrounds. Participants consistently emphasized the importance of culturally tailored programs to address the unique needs of these communities. Their insights highlighted the significance of incorporating both “surface structure” tailoring (e.g., language, dietary considerations) and"deep structure"tailoring into program design (e.g., family values, religious beliefs) [21, 22]. While providers advocated for deep cultural tailoring based on their clinical observations, our study design did not assess its actual effectiveness with patients. This distinction is important, as prior research demonstrates deeper cultural adaptation yields greater impact [22]. Future co-design with patients could validate whether provider-identified priorities align with survivor needs.

Our study supports previous research with Asian American cancer survivors, where HCPs report the need for culturally sensitive care to address stigma, and a recognition of differing communication styles [23]. Similarly, the healthcare professionals in our study recognized that cancer survivors from Asian backgrounds might downplay their symptoms, struggle to prioritize their individual needs over familial and community obligations, and exhibit reluctance to engage with healthcare services following cancer treatment. Addressing this “cultural hesitance” is a critical aspect of program design, as highlighted in several existing supportive care initiatives [24].

Our findings extend previous research highlighting the preference for supportive care approaches that addresses physical, psychological and social needs [25]. This aligns with established frameworks that advocating for integrated person-centred care that considers whole individual, and meets personal preferences [26]. HCPs indicated a holistic approach may incorporate mindfulness, exercise, and diet into a supportive care program. Consistent with previous research, “mindfulness” emerged as a valuable mental health strategy within cancer care [25]. This holistic approach resonates with a growing body of evidence demonstrating that these elements contribute to improved quality of life for cancer survivors [27, 28]. We emphasise that these preferences require validation through direct patient engagement to ensure alignment with survivor preferences and needs.

We found a strong preference among healthcare providers for the program to be offered by native speakers. This is consistent with previous studies where cancer survivors have expressed a preference for communicating in their native language [14]. Asian and Middle Eastern ethnic minority cancer survivors have reported a preference for passive participation [29], programs that are linguistically accessible may meet the identified lack of available information and support for people from CALD backgrounds [30].

Our findings suggest that interventions delivered within community settings, rather than medical or hospital environments, are more likely to be effective. These settings provide a sense of safety and familiarity, which is crucial for engagement and retention. This preference for community-based program delivery supports the model of care suggested by Tompkins et al. [31], which emphasizes the benefits of community engagement and peer support in enhancing the effectiveness of survivorship care.

Our study found HCPs expressed mixed preferences regarding delivery formats, with no clear consensus for face-to-face, hybrid, or online options. This aligns with previous research showing format preferences likely reflect individual circumstances [25]. However, systematic review evidence demonstrating better outcomes for face-to-face lifestyle interventions in cancer survivorship [28]. This this lack of consensus contrasts with systematic review data showing better outcomes from face-to-face lifestyle interventions in cancer survivorship [28]. This tension between efficacy and real-world implementation preferences warrants consideration in program delivery..

While Asian American cancer survivorship initiatives have increased [32, 33], there are limited Australian initiatives [33], and no published literature on survivorship programs for Arabic speaking cancer survivors. Smith et al. [11] noted a systemic lack of integrative oncology services tailored to ethnic minorities in Australia, reflective of a broader issue within healthcare service provision. In the pursuit of equitable cancer care, efforts need to be galvanized to engage ethnic minorities in Australia to understand their cancer survivorship experience and co-design programs that are culturally safe and championed by these at-risk communities. To implement these findings, healthcare services need to collaborate with community leaders to ensure that programs are culturally congruent and directly address the barriers CALD women face.

## Strengths and limitations

This study has several limitations. Firstly, while this qualitative study provides rich, context-specific insights into healthcare providers’ perspectives on culturally tailored supportive care, these findings are not intended to be generalisable. Rather, they offer in-depth understanding of the challenges and opportunities in developing programs for CALD populations within similar healthcare settings.

Secondly, our use of a pre-designed program for feedback, may have limited open-ended insights, and future research should consider more participatory methods.

Thirdly, our recruitment and interview methods have limitations. The small number of participants from different healthcare provider backgrounds in this study may not reflect the views of all groups. We did not use bilingual research assistants in our interviewers, this may have impacted cultural understanding. The recruitment methods employed which may also have influenced the study results. For example, the focus was on a specific region within Sydney.

Fourthly, the study did not include transcript review or participant validation of findings, which could have strengthened the credibility of the interpretations.

Finally, we have reported provider perspectives only, which may not fully capture patient experiences. While parallel research with survivors is underway (to be reported separately), this study’s value lies in its focus on implementation feasibility from the provider standpoint.

This study’s strength lies in its focus on healthcare providers experienced in survivorship care for CALD populations, offering practical and relevant insights. Semi-structured interviews enabled an in-depth exploration of culturally tailored program design and delivery needs. Recruitment from a diverse region like Western Sydney provided valuable context.

### Future research directions

Further research is planned to iteratively develop and adapt cancer survivorship programs for three culturally diverse groups based on this work and underpinned by the step-by-step approach developed by Costas-Muniz et al. [34]. The programs will incorporate the specific elements identified in this study, such as bilingual program delivery and community-based settings.

Studies could employ mixed-methods approaches to capture both the qualitative experiences of participants and quantitative data on health outcomes. Additional research would explore the impact of such interventions on long-term health outcomes, including quality of life, and measuring the cost-effectiveness of these culturally tailored interventions would provide valuable data to support policy changes and funding allocations.

## Conclusion

This study identifies healthcare providers'perspectives on key design elements for culturally tailored supportive care programs targeting CALD women with cancer, including preferences for delivery formats, content, and implementation strategies. These findings offer practical insights for developing culturally responsive care models within similar healthcare contexts. However, as this study exclusively reflects provider views, future research incorporating direct patient input is essential to validate these recommendations and assess their impact on health outcomes and equity.

## Supplementary Information

Below is the link to the electronic supplementary material.

Additional file 1: Focus Group/Interview Schedule for Health Care Providers

Additional file 2: Health Care Provider Thematic Analysis

## Data Availability

Raw data are not publicly available to preserve individuals’ privacy.

## Footnotes

1 Centrelink is an Australian Government agency that delivers social security payments to citizens and residents of Australia.
