# Supplementary material for: Supporting wellness after cancer treatment for women from Chinese, Vietnamese, and Arabic backgrounds: a qualitative study of healthcare provider views
Source: Support Care Cancer. 2025 Apr 17;33(5):394. doi: 10.1007/s00520-025-09417-6 (PMC12006199; doi:10.1007/s00520-025-09417-6)
Supplement: Supplementary file 1 — Additional file 1: Focus Group/Interview Schedule for Health Care Providers [file 520_2025_9417_MOESM1_ESM.docx]

**Focus Group/Interview Schedule for Stakeholders**

1. What type of support services do you provide for women who have completed cancer treatment?

*Probe for opinions on accessibility, appropriateness, sustainability and participation rates*

1. What has it been like to support women who have completed cancer treatment?

*Probe for concerns*

*Probe for those from Arabic, Cantonese, Mandarin and Vietnamese speaking backgrounds*

1. Do you believe these services can be improved?
2. What type of services do you personally think are needed to help these patients keep well and prevent cancer from coming back?
3. We are interested in developing a support program suitable for women from your community with cancer. There are many different ways that this can be provided. We would like to explore options for offering a “stepped care program”. What are your thoughts around this model?

SLIDE SHOWING THIS DIAGRAM AND EXPLANATION:

In a stepped care program, the needs of patients are matched to the intensity of the intervention, with more intensive interventions reserved for patients who do not benefit from low-intensity interventions, and considers patient preferences.


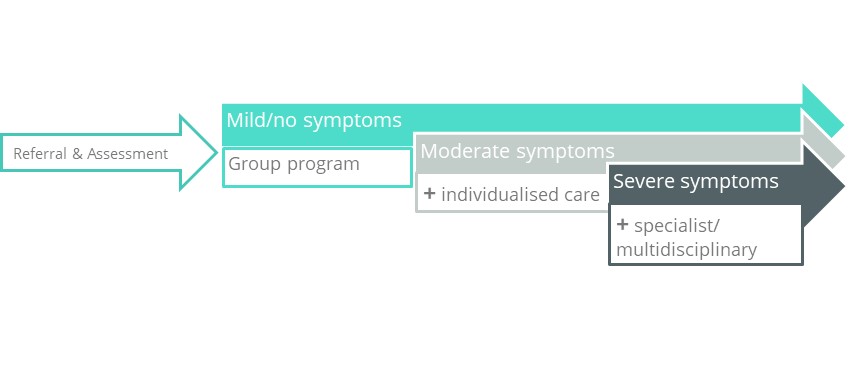


1. For those with moderate symptoms, where symptoms require more support, what might be the best way to offer this?
2. We would like to know the best way that this might be offered.

*Probe - What should it include (content)?*

*Probe - individualised assessment, nutrition, experiential sessions of mindfulness, yoga, tai chi, and exercise; assessment and treatment for side effects;*

*Probe - How and where should we offer this (mode, location): Use the internet for delivery, face-to-face, individual or group, community, home/family based etc*

*Probe - What sort of time commitment would be best (duration, frequency):* how often, how long

*Show slide with simple draft program – probe for feedback*

1. Would you be interested in being part of the delivery of the program?
2. How do you think such a program should be advertised?
3. What do you think would support or prove a barrier for women with cancer in using this support?

*Probe for facilitators/barriers*

*Probe for need for financial incentives*

1. Are there any cultural needs that you think require consideration?
2. How do you suggest we meet those needs?
