# Supplementary material for: Supporting wellness after cancer treatment for women from Chinese, Vietnamese, and Arabic backgrounds: a qualitative study of healthcare provider views
Source: Support Care Cancer. 2025 Apr 17;33(5):394. doi: 10.1007/s00520-025-09417-6 (PMC12006199; doi:10.1007/s00520-025-09417-6)
Supplement: Supplementary file 2 — Additional file 2: Health Care Provider Thematic Analysis [file 520_2025_9417_MOESM2_ESM.docx]

Health Care Provider Thematic Analysis

| **Themes and subthemes** | **Quotes from HCPs** |
| --- | --- |
| **Program Content** | |
| Holistic approach | “I’d like to have physical activity…emotional well-being... mindfulness and strategies for people and coping strategies… definitely nutrition. It's a big part of how people feel about themselves, but also not just that, but thinking outside the box and sort of having a bit more creativity, I guess in perhaps not just the traditional allied health and nursing and medical professions, but are there other more creative things, music therapy, other things like that that could be involved that just encompass the whole of someone.” [HCP4]  “The program would be about helping them understand how they can foster wellness, increase their vitality and increase their wellness to safeguard from future disease, rather than just focusing on the disease and learning how to live well with cancer rather than being a cancer patient or cancer survivor.” [HCP7]  "So from survivorship point of view we've got the programs we run…they're both for patients and carers and they also can have access to our programs. So oncology massage, acupuncture, yoga, meditation, gardening and so forth" [HCP6]  “And then we say we start saying, how are you? And they'll tell us the main symptoms, but they can't within that time. You know, follow ups are usually 15 minutes for me, 15 minutes for other people it could be shorter. And you know, there's only time to probably talk over the main one or two symptoms to examine and then talk about the scan results. That's it.” [HCP10] |
| Education, diet, exercise, psychosocial | “Well, I think I don't think it's [program content] really very much any different from the survivorship needs of the usual cancer population, having something, something like the [name of program] to transition them back to care, to life after cancer. You know, combination of education about this is what to expect. This is the elements of healthy living, how to eat healthy, how to exercise more.” [HCP11]  “Exercise and weight loss or minimising weight gain program would be good. And then some discussion around sexuality and body image and things like that after a lot of these women will have mastectomies. And from their medications have vaginitis and other issues. But again, I just want to say I don't really have a lot of time to address in my follow up appointments. I don't think I've routinely asked this. I think some of the GPs do.” [HCP10]  “I mean, I'm from a [name of CALD heritage] speaking background and for example that, you know, it's a very heavy carbohydrate diet.…I've had white rice every day of my life that no one has ever said that that's an issue. So yes, there's definitely, you know, cultural norms of diet that are not necessarily now what we think to be healthy.” [HCP12]  “I think diet is very important to patients when [they finish treatment. For some reason they're all interested in diet, so get dietician to come along and have a chat, maximum benefit…” [HCP5]  “So I've often found that just teaching some theraband exercises or some light weights with cans. And really getting THEM to do it and have a bit of a laugh and then interactive to then provide that on the spot feedback and coming back and then re-checking I think works really well. [HCP8]  "Empowerment absolutely. Very important because they come out of that feeling very disempowered...it's really important that they then feel empowered again and then repair their relationship with their body" [HCP7]  “need to mindful of the trauma, the psychological impact” [HCP9]  “..And that again worked well because it provided women with the same native language to come together and speak and share and have that time for listening and empathy” [HCP8] |
|  | ‘”.. can get a like a package, like a health information, the eating and you know a the friendly atmosphere with your friends who are the same situation and do you know..like a mind, a spirit, always lift up in this group” [HCP3]  “the education are really important to, to have to debunk all of that [misinformation]”. [HCP2]  “So discussion about recurrence and I guess acknowledging that that's common, that the fear of recurrence is common and I guess ways for them to be able to work through it and manage it” [HCP10]  “The thing that came up the most was the fear, the constant fear that it's going to come back. And that was something we were always addressing” [HCP7]. |
| **Program Delivery**  Setting | “I would do it in the community, in the community center, where they feel safe, where they feel that they won't be discriminated in any way and being delivered by people who are empathetic and who really understand the nuances of the cultural things of the group. So even the middle range people, I will still do it in the community environment. I will not do it in the hospital environment.” [HCP12]  “...about not being in that hospital setting because a lot of people, once they finish their treatment, they don't want to keep coming back to the hospital. And then there's all these other issues like parking and, you know, it's just people like to stay away as much as possible” [HCP 5]  “I think a combination because really, I think that you can't stick to one. What works for one person doesn't work for everybody. But I think in general you will get most people now using the internet. So I guess that's a very easy option. But I think some of those other things are definitely more, more built into their culture, they're more trusted and they're more familiar with those other avenues of doing things” [HCP13]  “I know from some recent research we've done, people just want easy access. So I think whether it's within the cancer center or whether it's in a community hall, I think looking at where it's placed. Is there parking and the timing helps to embed it for the patient” [HCP 8]  “Even group interaction. Sometimes zoom is also easier now. Now we can have the zoom link as well. It's so convenient now, especially if patients located in quite a big area is just a not applicable to be physically be in one location. So sometimes I feel that for certain exercise program maybe to form a group that might be a better way [HCP 2] |
| Stepped Care | “So I guess if you're a new person to the group, you may want a little bit more intervention. And then as you progress and you recover, you may not need as much intervention” [HCP13]  "Stratifying... that the people that need more support and help are put into different categories" [HCP12]  “We ran it as a group session where women were able to share, and they listened around, it was for breast cancer. …What happens with normal shoulder range of motion? What happens when there's a dysfunction. And obviously health literate language was used, but then women were able to share. We pause, we'd stop, we'd share and then we'd ask women to stand up and say, well let's have a look at your range of motion. We do it in a group.” [HCP8]  “So what you're proposing would be a good transition into the for these patients to transition to a program out in the community.” [HCP5_6] |
| Session timing | “I think somewhere between six weeks plus because I think by the time you get in and introduce what you're doing and then identify the needs and then offer the service, perhaps you're going to need at least six weeks to do and perhaps even more three months, I'm not sure. Six months? Six weeks to three months, perhaps” [HCP13] |
| Staffing | “It's very important that a coordinator or the facilitator should play a crucial role, because that facilitator needs to really understand the different, the cultural, and to be able to facilitate, to really understand each individual patient's need and to be able to engage with the different services they receive.” [HCP3] |
| Cost | “Free...That's what I think because most, most of the circumstance they can't go to work, I know, and their finances is very limited [HCP1]  “I feel it should be free. Yes. Yes, I think so. We have to promote government to provide some funding for this particular program.” [HCP2] |
| **Barriers and Enablers** | |
| Cultural safety | “Another pipe dream is a culturally specific survivorship clinic where they're seen by, there's all the right people in the one clinic, with caregivers and good interpreters.” HCP10  “So I think it works really well, and I think it links back because you're giving them a safe space and you're giving them time and they're like-to-like but there's differences, but we respect the differences but we're similar because we're all there for that same purpose” [HCP8]  “One thing is a safe and welcoming environment. That is the most essential thing, for a multicultural woman. And second thing is give them the opportunity to talk and share their stories with the people within the group [HCP12]  “I feel that should be in non-English speaking…So that can have some deep understanding. And also people might feel more connected immediately. And also that a facilitator need to have quite a bit of training or they already have some decent experience in not just the medical knowledge, but also cultural awareness themselves” [HCP2] |
| Cultural norms & myths | “Yeah. I mean, is it is all going to be sweeping generalizations, I have to say. But I tend to find that Cantonese speaking and Vietnamese speaking patients often don't give me a lot. It's a lot of like I'm good thank you, [name of job title] like everything's fine kind of thing... ..still that really was so respectful of the doctor. I'm not going to sort of take up their time kind of thing”. [HCP10]  “She had breast cancer. She went and had the mastectomy done without her husband's knowledge. And to date, she still hasn't told her husband she had breast cancer.” [HCP12]  “They don’t want anyone outside their family to know what’s going on…So they don't want to talk to or have any further engagement. And the other issue would be they worried about if if they attend to most services. So does it mean that actually they are not cured?” [HCP2]  “So I guess comprehension and language would be one thing. And I think the other part of that is for all of those different cultures there are in my experience, the things that I've seen when I've looked after these women, is that there are different cultural beliefs and backgrounds to the way that they relate to the medical profession. What I'm trying to probably say, I'll break that down a little bit, is that when I see a woman and she may be with her husband, if she's from a perhaps a Vietnamese background, she may be a little bit more shy and reserved about what she says. And perhaps the male partner may be the person that drives some of the conversation. I think sometimes that happens too in Middle Eastern, and I guess too the Middle Eastern woman in my experience tends to be a little bit more emotional. She's more she's more, not as transparent. She's will allow her emotion and her emotions to be seen. She doesn't guard them. She'll say exactly how she feels and she will perhaps cry and show those emotions in front of people. Whereas those other cultures tend to be very shy and they try not to perhaps show those emotions in front of people.” [HCP13] |
| A skilled and trusted facilitator | “we can go through the oncologist. Hospital. You'll have to let them know. Each hospital has social worker” [HCP2]  “So I did a lot of work on boundaries, both verbally as well as experientially, like having them put something down on the ground where their boundary is. And learning to identify and learning how to communicate their boundaries in an assertive way rather than passive aggressive, and they didn't know that there was a third option called assertive.” [HCP7] |
| Language | “But I see that, when they find, you know, radiation therapists or chemo nurses who speaks the language, they trust them a lot more.” [HCP11]  “some culturally appropriate dancing or other types of physical activity. I guess just kind of education, the usual kind of survivorship programs delivered in their first language would be great” [HCP11]  “And that again worked well because it provided women with the same native language to come together and speak and share and have that time for listening and empathy” [HCP8]  “I feel that should be in non English speaking…So that can have some deep understanding [HCP2]. |
| Inclusion of Families and Carers | “we had a family cancer forum. We invited, the whole community with the kids and everyone with food provided a Q&A session with all the specialists and things on stations, anybody can ask any questions” [HCP 12];  “So I think it's important to involve the carers as well and I think they're a bit forgotten about at times. They just do all the hard work and all that stress, worrying about appointments and getting people in treatment and so forth. But they've been neglected when it comes to our services.” HCP5 |
| Raising awareness about the program | “But there's a lot of community groups, cultural groups on Facebook and so on that link up that way too. I'm sure there's ways to get the word out, but it has to be done in a proper way to be able to access certain communities” [HCP 10]  “So if every one of those health professionals knew about it and advertised or word of mouth, then that would be one avenue, and that would be very close to the patient, because it would be in that cancer treatment area, treatment world. And then I guess Medicare, GP, all that aftercare sits outside of a hospital and outside of treatment. So follow up with GPs and care like that” [HCP13]  “Well is there benefit of doing it on different CALD radios as well? You know, their own networks, you know, women might listen to their own local radio, their own local papers” [HCP8]  “I think they should get it from their oncologists. Just before the oncology treatment finishes or start talking in midway. You know, when you when you finish the treatment, this is something that you can do to take care of yourself, I think that should be drummed in” [HCP2] |
| Financial | “It has to be free. If it is not free, these women will not come, and nobody is going to pay to attend a survivorship program.” [HCP12] |

-
